# Book review: Manual of Central American Diptera, Volume 1. B. V. Brown, A. Borkent, J. M. Cumming, D. M. Wood, N. E. Woodley, and M. Zumbado (Eds) (2009) National Research Council of Canada, Ottawa, 714 pp.

**DOI:** 10.3897/zookeys.52.541

**Published:** 2010-07-30

**Authors:** Atilano Contreras-Ramos

**Affiliations:** Instituto de Biologia, UNAM, Depto. de Zoologia, Apdo. Postal 70-153, 04510 Mexico, DF, MEXICO

As an undergraduate biology major in northern Mexico I do remember my first attempts at keying out some aquatic insects I had collected for a class project. That was in the early 80’s, still some of the manuals for North America (United States and Canada) worked out fairly well. Nonetheless, soon I realized that even for Mexico, close to the United States yet with a strong Neotropical component, insect identification was a difficult and somewhat uncertain task. As a Mexican entomologist I have a general perception about well-studied faunas (e.g., western Europe, North America, Australia), but if I had to select a well-known taxon I would probably pick Diptera. And if I had to name a diverse and complex fauna, where species would be hard to identify, most likely I would say South America and the rest of the Neotropics.

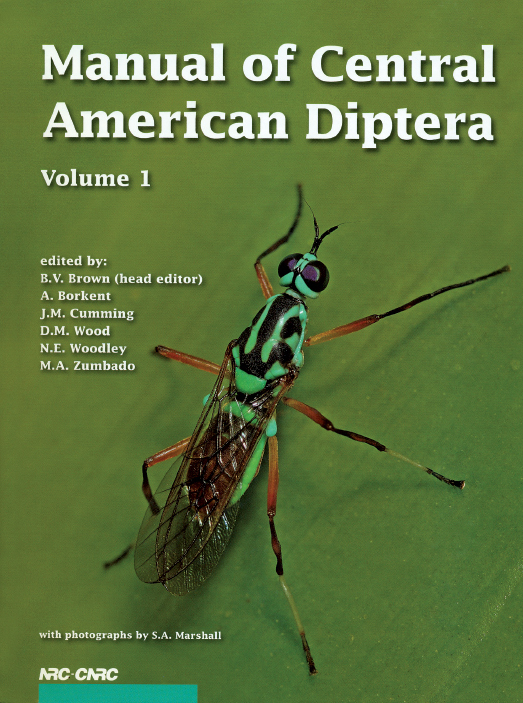



However, things are changing for us in Latin America, and for good. A solid tradition of dipterology in South America is now getting organized to produce a fast-publication journal, Neotropical Diptera (Amorim and Papavero 2008). This goes side by side with the goal of having a Manual of Neotropical Diptera, a challenging job. The Neotropics hold about 25,000 described fly species (Amorim and Papavero 2008), which would be about 21% of the world Diptera fauna of 120,000 species (Grimaldi and Engel 2005), a pretty respectable figure. Meanwhile, a subsection of Neotropical fly diversity has become manageable in the form of a Manual of Central American Diptera (MCAD), an intended two-volume set, of which the second one will become available any time.

The MCAD will treat 106 families in the region, of which 42 have been included in volume 1. The area of coverage for the manual is circumscribed from Panama to Guatemala and Belize, plus tropical Mexico (the Yucatan, southeastern Mexico, through both coastal lines, the Balsas Depression south of the Volcanic Axis, and the tip of the Baja California Peninsula). Volume 1 includes seven condensed introductory chapters, corresponding to an introduction, adult morphology and terminology, natural history, economic importance, phylogeny, key to families for adults, and key to families for larvae. A total of 45 specialists authored the different chapters, of which only 6 are based in Latin America; the rest are based in Australia (1), Europe (12), United States and Canada (25). This speaks of the need to stimulate entomology in Latin America, a role this book for sure will have to a decent share.

As its ancestor, the 3 volume series Manual of Nearctic Diptera (MND; McAlpine et al. 1981, 1987, 1989), the MCAD has excellent illustrations, many taken from the MND. Chapter 2 on adult morphology is a delicacy for current and potential dipterists. It is organized in the form of seriated glossaries, following a body region arrangement. This seems to be a more straightforward fashion of presenting complex information that might be arid in regular prose. There is no chapter on larval morphology, however chapter 7 on a key to families for larvae might cover some of that need. The preceding chapter on a key to families for adults is complemented at the end with nice color photographs of habitus of each fly family.

The chapter on phylogeny is a succinct, yet quite complete synthesis of the relationships of Diptera major groups and families. It sacrifices, of course, much of the detail in volume 3 of the MND, such as the extensive character discussion. Both treatments are fairly concordant, however some differences are more or less evident in the MCAD (e.g., Nematocera is explicitly paraphyletic as Brachycera is sister to Anisopodidae; phylogeny of Brachycera is better resolved and it contains Schizophora, so it is explicitly paraphyletic).

The title of each family chapter includes the taxon Latin name followed by common names in English and Spanish. Each chapter is to be praised. They include sections on diagnosis, biology, classification, identification, a key to genus, and synopsis of the fauna. A strong effort to synthesize available information on a previously mostly untreated fauna is worth recognizing, especially for highly diverse groups. The emphasis is on adults, however most chapters include at least some illustrations of immatures, some include keys to larvae (e.g., Simuliidae), others include expansion of coverage area (e.g., Nearctic Mexico, in Simuliidae and Stratiomyidae).

Taxonomic chapters correspond to nematocerous families (23) and lower brachycerous families (19). Of the former, one is an unplaced group within Sciaroidea (the Ohakunea group), and in the latter, the Mythicomyiidae is recognized as a distinct family out of Bombyliidae. Many families are shared with the Nearctic fauna, yet several are not included in the MND (i.e., the nematocerous Ditomyiidae, Diadociidae, Keroplatidae, Lygistorrhinidae, and the brachycerous Pantophthalmidae), some are surprisingly present (e.g., Ptychopteridae). This manual will for sure increase noticeably the amount of works on Diptera species in the region, not only taxonomic, but also ecological and biological. Volume 1 is a more than welcome addition to the library of any dipterist, broad minded entomologist, or naturalist, and should be present in university libraries throughout the region. Meanwhile, volume 2 is anxiously awaited for the completion of the set. This book may be acquired through the online bookstore of the National Research Council of Canada (http://pubs.nrc-cnrc.gc.ca/eng/books/browse/list-0-0.html).

## References

[B1] Amorim DS Papavero N (2008) Editorial. A journal for the systematics and biogeography of Neotropical Diptera, 250 years after the publication of the tenth edition of the Systema Naturae. Neotropical Diptera 1:1-5

[B2] Grimaldi D Engel MS (2005) Evolution of the Insects. Cambridge University Press, xv + 755 pp.

[B3] McAlpine JF Peterson BV Shewell GE Teskey HJ Vockeroth JR Wood DMEds (1981) Manual of Nearctic Diptera, vol. 1. Research Branch, Agriculture Canada, Monograph No. 27, Ottawa, 674 pp.

[B4] McAlpine JF Peterson BV Shewell GE Teskey HJ Vockeroth JR Wood DMEds (1987) Manual of Nearctic Diptera, vol. 2. Research Branch, Agriculture Canada, Monograph No. 28, Ottawa, pp. 675–1332

[B5] McAlpine JF Wood DMEds (1989) Manual of Nearctic Diptera, vol. 3. Research Branch, Agriculture Canada, Monograph No. 32, Ottawa, pp. 1333–1581

